# Assessment of Patients’ Perception of Telemedicine Services Using the Service User Technology Acceptability Questionnaire

**DOI:** 10.5334/ijic.2219

**Published:** 2016-06-01

**Authors:** Claudio Dario, Elena Luisotto, Enrico Dal Pozzo, Silvia Mancin, Vassilis Aletras, Stanton Newman, Lorenzo Gubian, Claudio Saccavini

**Affiliations:** Chairman of Arsenàl.IT Veneto’s Research Centre for eHealth Innovation, Treviso, Italy; General Manager of the Padua Teaching Hospital, Padova, Italy; Clinical Psychologist of Arsenàl.IT Veneto’s Research Centre for eHealth Innovation, Treviso, Italy; Anthropologist of Arsenàl.IT Veneto’s Research Centre for eHealth Innovation, Treviso, Italy; Project Manager of Arsenàl.IT Veneto’s Research Centre for eHealth Innovation, Treviso, Italy; Associate Professor of Health Care Management & Health Economics, Department of Business Administration, University of Macedonia, Thessaloniki, Macedonia, Greece; Professor in Health Psychology and Dean of School of Health Sciences, City University, London, UK; Technical Manager of Veneto Region Health Information System, Veneto Region, Venice, Italy; Technical Manager of Arsenàl.IT Veneto’s Research Centre for eHealth Innovation, Treviso, Italy

**Keywords:** telemedicine, acceptability, chronic, perception, integrated care, HTA

## Abstract

**Introduction:**

The purpose of this paper is to assess if similar telemedicine services integrated in the management of different chronic diseases are acceptable and well perceived by patients or if there are any negative perceptions.

**Theory and methods:**

Participants suffering from different chronic diseases were enrolled in Veneto Region and gathered into clusters. Each cluster received a similar telemedicine service equipped with different disease-specific measuring devices. Participants were patients with diabetes (n = 163), chronic obstructive pulmonary disease (n = 180), congestive heart failure (n = 140) and Cardiac Implantable Electronic Devices (n = 1635). The Service User Technology Acceptability Questionnaire (SUTAQ) was initially translated, culturally adapted and pretested and subsequently used to assess patients’ perception of telemedicine. Data were collected after 3 months and after 12 months from the beginning of the intervention. Data for patients with implantable devices was collected only at 12 months.

**Results:**

Results at 12 months for all clusters are similar and assessed a positive perception of telemedicine. The SUTAQ results for clusters 2 (diabetes), 5 (COPD) and 7 (CHF) after 3 months of intervention were confirmed after 12 months.

**Conclusions:**

Telemedicine was perceived as a viable addition to usual care. A positive perception for telemedicine services isn’t a transitory effect, but extends over the course of time.

## Introduction

Healthcare systems are dealing with an ageing population affected by chronic disease and social care needs that will lead to an increase in health service demands. Such a phenomenon could result in a lack of resources and undermine healthcare services to patients [1,2]. In this regard, telemedicine, in its generic sense so as to include telehealth and telemonitoring, is seen as a potential integrated care solution to this problem as it could support people in their own homes, improving the quality of health service provision, potentially encouraging the self-management of health problems and increasing the cost-effectiveness of care for people with long-term conditions [3,4,5,6]. Patient perception is an important step in the evaluation of telemedicine services, as patient acceptability and satisfaction are relevant to any potential roll out of these services and commonly used indicators for measuring quality in health care [7,8,9,10].

The aim of the study was to measure acceptability in the conditions under study and to examine whether it changed over time. In this paper we specifically assess patients’ perception of telemedicine services in the RENEWING HEALTH [11,12] European project. This set out to deploy a large-scale real-life pilot for the evaluation of innovative telemedicine services for patients with chronic disease using a common rigorous assessment methodology called MAST (Model for Assessment of Telemedicine) [13,14].

The Veneto Region Social-Health plan aims to focus both on integrating health and social care and on the integration between hospitals and primary care. The integrated management of chronic patients in Veneto provides the implementation of new care models characterized by a multidisciplinary approach that ensure continuity of care and promote the dissemination of clinical pathways, with a consistent and coordinated use of resources. A specific commitment is focused on the implementation and deployment of telemedicine services for fragile patients with limited access to healthcare services [15].

## Theory and Methods

The “Renewing Health” project started in February 2010 and ended in December 2013 and assessed a panel of about 7,000 patients selected from the nine countries involved in the project. In each country, participants with different chronic diseases were equipped with specific telemedicine services and enrolled in 10 specific clusters of patients. Each country assessed at least one cluster of patients.

In this paper, we present the results of 2,118 patients out of total of 3,332 patients that participated in “Renewing Health” wider randomized controlled clinical trial in the Veneto Region. Participants were enrolled in 4 specific clusters: Cluster 2 (diabetes) – type 2 diabetes, HbA1c > 53 mmol/mol (7.0% according to NGPS); Cluster 5 (COPD) – chronic obstructive pulmonary disease (COPD), GOLD Class III-IV; Cluster 7 (CHF) – chronic heart failure (EF < 40% or EF > 40% plus BNP > 400 or plus NT-proBNP > 1500), discharge from hospital after acute heart failure in the previous three months; Cluster 8 (PM/ICD) – patients with implantable devices (pacemaker – PM and implantable cardiac defibrillator – ICD) [16].

All participants, both intervention patients and control patients, had to meet the following inclusion criteria to gain entry to the project: age ≥ 18 years (except Cluster 7 ≥ 65 years); being cognitively able to participate; being able to use the equipment (alone or assisted); being able to answer the questionnaires in the native language; absence of severe comorbidity prevalent on specific illnesses with a life expectancy < 12 months; not enrolled in another trial; being able to provide written informed consent to participate in the trial. Nine local health authorities (LHA) in the Veneto Region participated in the study. Patients were selected by all participating hospital departments following hospital discharge, based on outpatient visits, or by screening electronic healthcare records.

The “Renewing Health” project was a randomized controlled trial with a telemedicine intervention group and a control group treated in usual care. Discharged patients entered in usual care. The acceptability questionnaire was only administered to the intervention group. The SUTAQ questionnaire was administered after 3 and 12 months to the intervention groups in Clusters 2-5-7, in this paper are presented only the results of patients that answered both at 3 and 12 months. In Cluster 8 patients answered only after 12 months. In the Veneto Region, the SUTAQ was administrated by telephone via operators of the Regional eHealth Centre (Clusters 2-5-7) and by Arsenàl.IT anthropologists that took part in the Renewing Health project (Cluster 8). Patients answered the questionnaire in about 15 minutes.

To assess the acceptability of different telemedicine services, the results from different clusters are analysed separately and then compared. The intervention in the Veneto Region [17,18,19,20] centre around two services: Telecare and Telehealth. In the telehealth group patients used the provided device at home to measure their vital signs as appropriate to their condition. These were carried out in accordance with the monitoring plan agreed with the reference clinician on enrolment. The monitoring plan shows the days and times at which the patient is expected to perform the measurements. Telemonitoring devices used by the patient collect data and send them to the gateway device which transmits the data to the Regional eHealth Centre. At this centre a group of operators with refer to the monitoring plan defined by the reference clinician, checks the data sent by the patient. Should the clinical parameters be out of a specified range, the telemonitoring software produces an alarm which triggers the operator to intervene and manage according to the standard protocol. In the Tele-care Service the patient uses the emergency button provided to trigger an alarm in the case of an emergency (social or health). The Centre’s operators periodically called patients to monitor their condition.

Patients enrolled in cluster 8 were not followed by the Regional eHealth Centre and data were reviewed by the reference clinic. PMs and ICDs are provided with a small antenna able to send data recorded by the PM/ICD periodically to a home external gateway [21]. Transmission could occur at pre-established intervals, or in the case of adverse events (related to the status of the patient or to the running of the electrocatheter-device), without a particular intervention on the part of the patient. The gateway forwards data to different external provider servers depending on the ICD/PM vendor. Data stored in the specific vendor server are available for health professionals through a web-browser that allows them to check the patients’ vital signs. A professionally trained nurse is placed in charge of checking RM data as a primary filter. As regards observations of critical occurrences or unclear data interpretation, data are then submitted to the physician who decides on the optimal decision to take. Recorded data are reviewed only during health professionals’ normal working hours.

### Description of the questionnaire

To determine which instrument to use to assess acceptability a literature review was performed. The Service User Technology Acceptability Questionnaire (SUTAQ) was adopted to assess patients’ perception. This questionnaire is based on a literature review and on testing in qualitative studies. The questionnaire was used in the Whole System Demonstrator project (WSD), which included approximately 3,000 patients [22].

Patients’ perception was assessed using the Service User Technology Acceptability Questionnaire (SUTAQ) [23,24,25]. The questionnaire was translated from English to Italian using the forward backward translation method and tested for ambiguities in the translation using 15 individuals in order to ensure cross-cultural comparability of the questionnaire versions [26]. The questionnaire consists of 22 items divided into six different subscales. The “Enhanced care” subscale involves items regarding patients’ concerns about health status, their perception of active involvement, recommendations to people in a similar condition, and perceptions of enhanced care; the “Increased accessibility” subscale includes questions about patients’ perception of time saving, of increased access to care, of health improvement and of easier contact with professionals; the “Privacy and discomfort” subscale consists of items regarding patients’ concerns about privacy and their perception of discomfort; the “Care personnel concerns” subscale includes questions about patients’ perception of continuity of care and concerns related to personnel involved in the service; the “Kit as substitution” subscale includes items regarding patients’ concerns about health status and their perception of the service as a substitute for regular care and face-to-face consultations; the “Satisfaction” subscale involves questions about patients’ satisfaction and their understanding of telemedicine services. The wording of the 22 items in the 6-point Likert scale questionnaire is both positive and negative, thus reducing related biases. The final result of each subscale indicates the degree of average internal agreement to it (6 = strong agreement and 1 = strong disagreement). The intermediate value 3.5 has to be considered as point of neutrality. The results of two subscales, “Privacy and discomfort” and “Care personnel concerns”, are inverted, therefore a low value in these subscales reflects a positive perception of telemedicine with regard to these two aspects of the service.

### Statistical method

For all clusters, except for cluster 8, the values of the intervention group for each cluster at 3 and 12 months were compared, and in performing the Wilcoxon Matched Pairs test (data were not normally distributed according to Shapiro and Wilk’s W test) the dependency of the data was considered. The effects of explanatory variables (gender, age and education) on multi-item scale scores were estimated by running classical linear regressions. No formal schooling plus less than primary education were used as the reference category and were excluded from the regression equation.

## Results

### Participant flow

Participant flow refers to “Renewing Health” wider randomized controlled trial, that included a control group that didn’t receive an allocated intervention and therefore didn’t answered the SUTAQ.

Cluster 2 (diabetes): 499 patients were assessed for eligibility, 200 were excluded as follows: 170 declined to participate, 21 didn’t meet the inclusion criteria, 9 for other reasons. 299 patients were randomized, 92 were allocated to control group; 207 patients were allocated to intervention, 27 didn’t receive an allocated intervention for the following reasons: 17 for technical difficulties, 10 for patient’s or relative’s will. 180 patients received an allocated intervention; 17 patients were lost to follow up (9.4%) as follows: 5 deaths (2.8%), 7 due to the patient’s or relative’s will (3.9%), 3 patients could not be reached at the expected time (1.6%), 2 for other reasons (1.1%). At 3 months, 167 questionnaires were submitted (92.8%); at 12 months, 163 questionnaires were submitted (90.6%).

Cluster 5 (COPD): 458 patients were assessed for eligibility, 124 were excluded as follows: 92 didn’t meet the inclusion criteria, 25 declined to participate and 7 for other reasons. 334 patients were randomized, 104 were allocated to control group; 230 patients were allocated to intervention, 19 didn’t receive an allocated intervention for the following reasons: 9 for patient’s or relative’s will, 7 for technical difficulties, 1 patient died, 1 was transferred to nursing home and 1 for other reasons. 211 patients received allocated intervention; 31 patients were lost to follow up (14.7%) as follows: 25 deaths (11.8%), 3 due to the patient’s or relative’s will (1.4%), one was transferred to a nursing home (0.5%), one patient could not be reached in the expected time (0.5%), one for other reasons (0.5%). At 3 months, 203 questionnaires were submitted (96.2%); at 12 months, 180 questionnaires were submitted (85.3%).

Cluster 7 (CHF): 419 patients were assessed for eligibility, 80 were excluded as follows: 56 didn’t meet the inclusion criteria, 12 declined to participate and 12 for other reasons. 339 patients were randomized, 110 were allocated to control group; 229 patients were allocated to intervention, 39 didn’t receive an allocated intervention for the following reasons: 17 for patient’s or relative’s will, 14 patients died, 6 for technical difficulties, 1 was transferred to nursing home and 1 for other reasons. 190 patients received the allocated intervention; 50 patients are lost to follow up (26,3%) as follows: 38 deaths (20%), 5 due to the patient’s or relative’s will (2.6%), one was transferred to a nursing home (0.5%), one due to technical difficulties (0.5%), 3 patients refused to answer the questionnaire (1.6%) and 2 patients could not be reached in the expected time (1.1%). At 3 months 166 questionnaires were submitted (87.4%); at 12 months, 140 questionnaires were submitted (73.7%).

Cluster 8 (PM/ICD): 2138 patients were assessed for eligibility, 37 were excluded as follows: 17 declined to participate, 9 didn’t meet the inclusion criteria and 11 for other reasons. 2101 patients were enrolled, 230 were allocated to control group. 1871 patients received allocated intervention; 149 patients were lost to follow up (8%), 111 deaths (5.9%), 12 due to the patient’s or relative’s will (0.7%), 15 due to technical difficulties (0.8%), 11 for other reasons (0.6%); 87 patients couldn’t be reached in the expected time (4.6%). At 12 months, 1635 questionnaires were submitted (87.4.%).

### Baseline data

The mean age of the intervention sample was between 72 years (cluster 8) and 79 years (cluster 7). The majority of participants were male, with a percentage ranging between 55% (cluster 2) and 70% (cluster 5 – cluster 8). Most participants had received less than a secondary school education, with a percentage ranging between 52% (cluster 8) and 72% (cluster 2). Most of the sample was living with an adult, with a percentage ranging between 80% (cluster 7) and 88% (cluster 8). Most participants enrolled in cluster 2 (61%) and cluster 8 (72%) reported not receiving any health care assistance at home, with a minority of participants in cluster 5 (35%) and cluster 7 (44%) stating that they were receiving health care assistance at home. All baseline data present similar characteristics (age, gender, education, living with an adult) except for the percentage of patients that stated that they were receiving assistance at home. Further baseline data are available in Table 1.

**Table 1:** Baseline data for all clusters.

Measurement	CLUSTER 2 (DIABETES) Intervention	CLUSTER 5 (COPD) Intervention	CLUSTER 7 (CHF) Intervention	CLUSTER 8 (PM/ICD) Intervention

**Sample size (n)**	163	180	140	1635
**Average age (years)**	73	75	79	72
**Men (gender)**	90 (55%)	125 (70%)	85 (61%)	1133 (70%)
**Female (gender)**	73 (45%)	55 (30%)	55 (39%)	497 (30%)
**Education**				
No formal schooling	3 (2%)	1 (1%)	0 (0%)	6 (0%)
Less than primary school	8 (5%)	16 (9%)	15 (11%)	145 (9%)
Primary school	106 (65%)	96 (53%)	76 (54%)	703 (43%)
Secondary school	28 (17%)	34 (19%)	17 (12%)	354 (22%)
High school	15 (9%)	24 (13%)	24 (17%)	302 (18%)
College/University	3 (2%)	8 (4%)	6 (4%)	96 (6%)
Post graduate degree	0 (0%)	0 (0%)	1 (1%)	16 (1%)
Missing answer	0 (0%)	1 (1%)	1 (1%)	13 (1%)

**Is there at least an adult that lives with you?**				

Yes	142 (87%)	153 (85%)	112 (80%)	1444 (88%)
No	20 (12%)	26 (14%)	28 (20%)	158 (10%)
Missing answer	1 (1%)	1 (1%)	0 (0%)	33 (2%)

**Is there anyone who assists you at home?**				

No	99 (61%)	63 (35%)	61 (44%)	1173 (72%)
Relative	62 (38%)	105 (58%)	62 (44%)	398 (24%)
Caregiver	1 (1%)	8 (4%)	11 (8%)	42 (3%)
Private nurse	0 (0%)	0 (0%)	0 (0%)	0 (0%)
Integrated Home Care	0 (0%)	0 (0%)	1 (1%)	1 (0%)
Other	1 (1%)	1 (1%)	5 (4%)	14 (1%)
Missing answer	0 (0%)	3 (2%)	0 (0%)	7 (1%)

### SUTAQ Results

Results of SUTAQ are presented in Table 2.

**Table 2:** SUTAQ results for clusters 2, 5, 7 at 3 and 12 months after the intervention and results for cluster 8 at 12 months. Two subscale, “Privacy and discomfort” and “Care personnel concerns”, have inverted scores thus a low value implies a positive view towards these aspects of telemonitoring.

	CLUSTER 2 (DIABETES) N = 163 90.6% on target			CLUSTER 5 (COPD) N = 180 85.3% on target			CLUSTER 7 (CHF) N = 140 73.7% on target			CLUSTER 8 (PM/ICD) N = 1635, 87,4% on target

Subscale	Intervention after 3 months	Intervention after 12 months	P value (< 0.05)	Intervention after 3 months	Intervention after 12 months	P value (< 0.05)	Intervention after 3 months	Intervention after 12 months	P value (< 0.05)	Intervention after 12 months
							
	Median (interquartile range)	Median (interquartile range)		Median (interquartile range)	Median (interquartile range)		Median (interquartile range)	Median (interquartile range)		Median (interquartile range)

**Enhanced care**	5.4 (5.2–5.8)	5,8 (5,2–6)	**<0.001**	5.4 (4.8–5.8)	5.6 (5–6)	**0.02**	5.6 (5–5.85)	5.8 (5.2–6)	**0.05**	5.8 (5.4–6)
**Increased accessibility**	5 (4.5–5.25)	5 (4,75–5,5)	**<0.001**	5 (4–5.5)	4.75 (4.25–5.25)	0.26	5 (4.5–5.25)	5 (4.45–5.5)	0.37	5.5 (5–5.75)
**Privacy and discomfort**	1 (1–1.25)	1 (1–1,25)	0.79	1 (1–1.25)	1 (1–1.75)	0.14	1.25 (1–2)	1 (1–1.25)	**<0.001**	1 (1–1)
**Care personnel concerns**	1 (1–1.66)	1,66 (1–2,33)	**<0.001**	2 (1.33–2.66)	2.33 (1.33–2.3)	0.39	2 (1–2.66)	1.66 (1–2.33)	0.34	1 (1–1.33)
**Kit as substitution**	3 (2.66–3.33)	2,66 (2–3)	**<0.001**	2.66 (2–3.33)	2.33 (2–3)	0.20	2.66 (2.33–3)	2.66 (2–3)	0.50	2.33 (2–2.66)
**Satisfaction**	6 (5.66–6)	6 (5,66–6)	0.91	5.66 (5.66–6)	6 (5.66–6)	**0.01**	6 (5.33–6)	6 (5.66–6)	**0.05**	6 (5.66–6)

**Figure 1:**
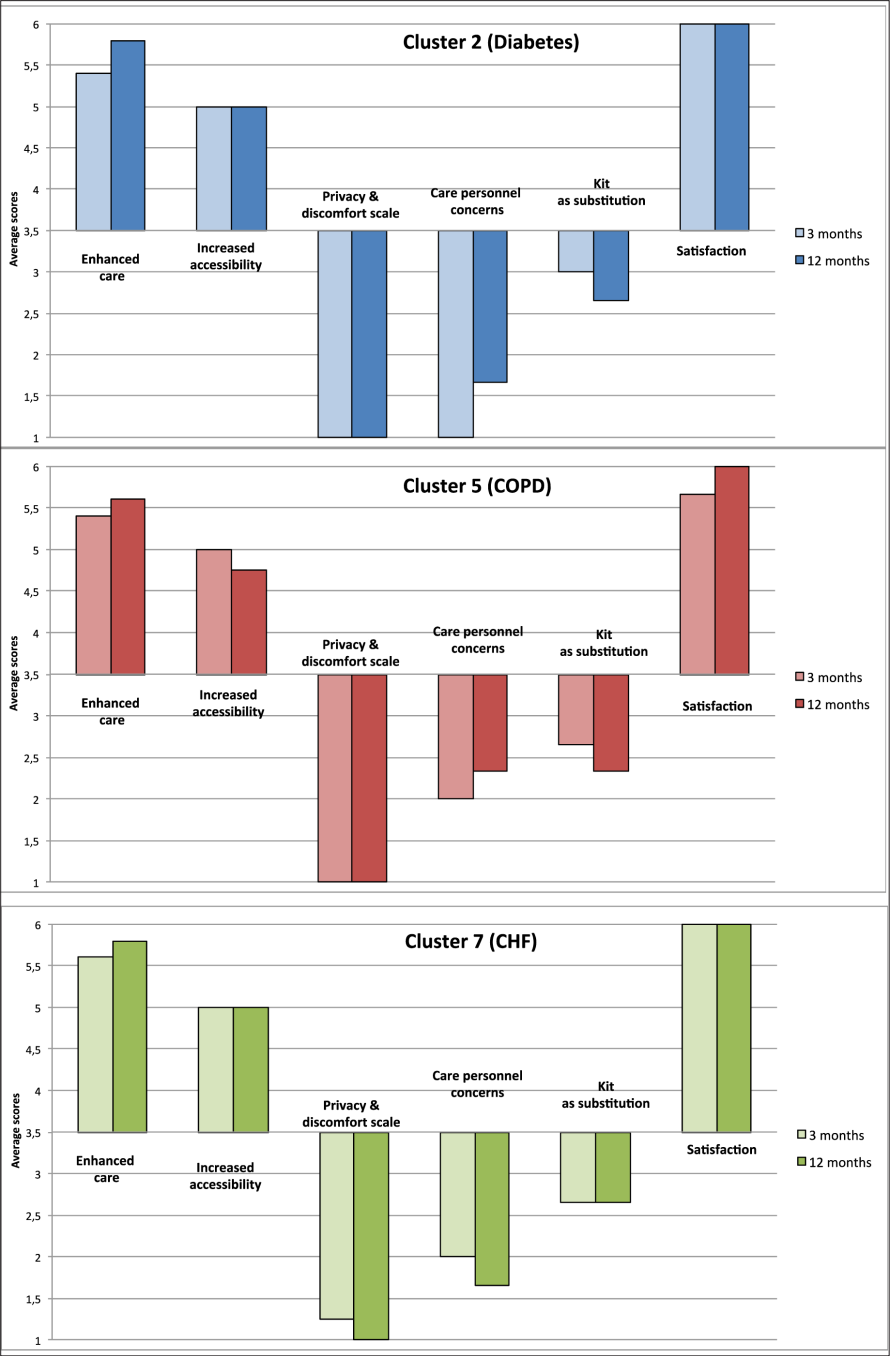
SUTAQ results at 3 and 12 months for clusters 2, 5, 7.

**Figure 2:**
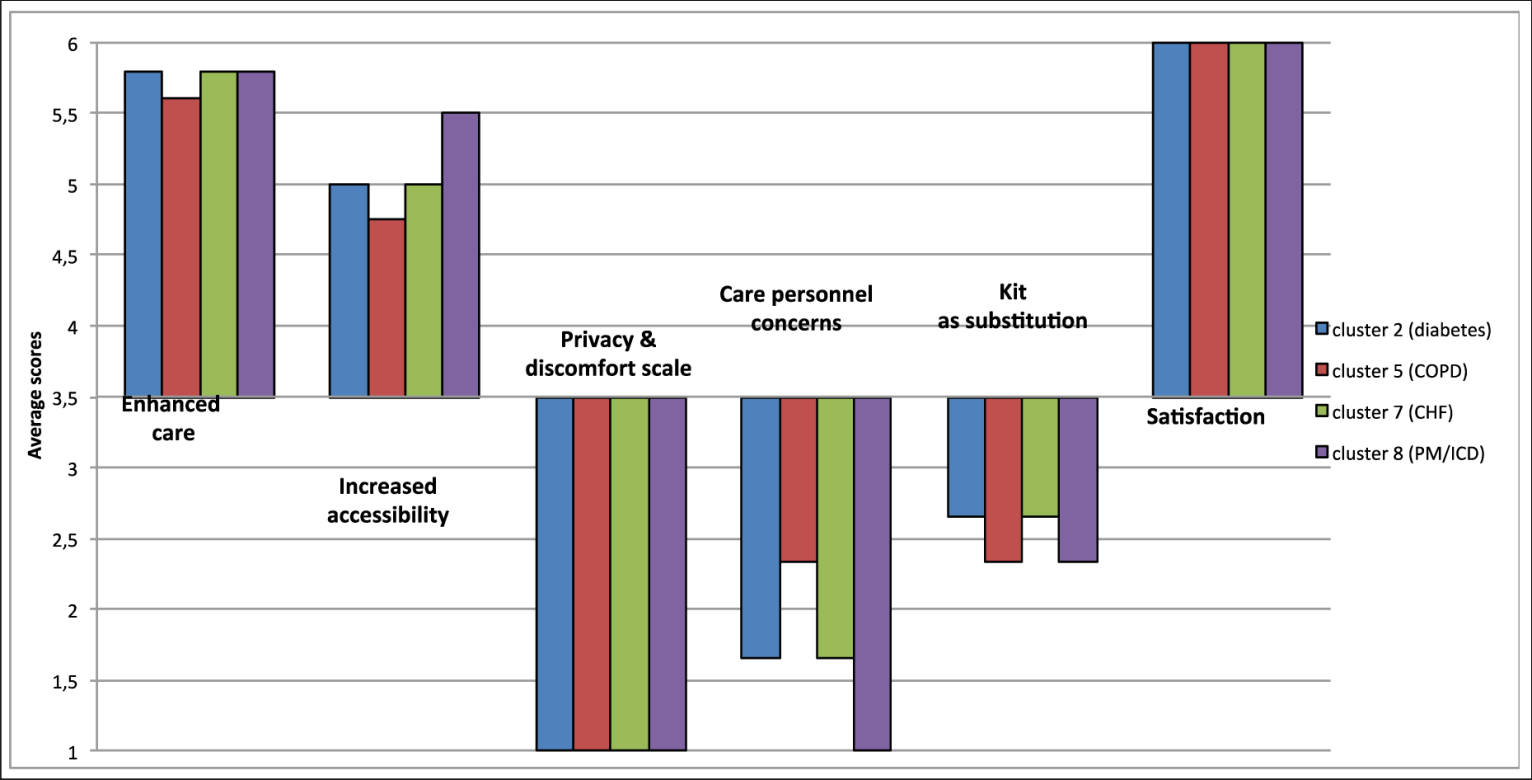
SUTAQ results at 12 months for all clusters.

The SUTAQ results for clusters 2 (diabetes), 5 (COPD) and 7 (CHF) after 3 months of intervention indicated a positive perception of patients towards telemedicine and this continued at the 12 months assessment (see Table 2 and Figure 1). Results at 12 months for cluster 8 (PM/ICD) are similar to the results of the other clusters (see Figure 2).

Three subscales, “Enhanced care”, “Satisfaction” and “Increased accessibility” indicated a high degree of acceptability of the service in all clusters. Results of the “Enhanced care” subscale increased significantly between 3 and 12 months in cluster 2 (p < 0.001), in cluster 5 (p = 0.02), and in cluster 7 (See Table 2). The results of the “Satisfaction” subscale increase significantly after 12 months of intervention for cluster 5 (p = 0.01), and cluster 7 (p = 0.03) (See Table 2). Results of the “Increased accessibility” subscale are high for all clusters, with patients reporting an increased level of accessibility due to the telemonitoring service at 12 months in cluster 2: (p < 0.001).

Results of the “Privacy and discomfort” subscale are low for all clusters, implying that patients weren’t concerned about privacy issues, thus they had a positive view towards these aspects of telemonitoring. Patients enrolled in cluster 7 had low assessments at 3 months and these decreased significantly at 12 months.

Results of the “Kit as substitution” subscale for all clusters was between 2 and 3 indicating that patients only mildly disagreed that the telemedicine service could act as a substitute of usual care. Cluster 2 showed a small but significant reduction on this dimension (p < 0.001).

Concern about the kit and care personnel was low for all clusters suggesting that participants weren’t concerned about this issue and they had a positive view towards these aspects of telemonitoring. Although low it is of note that patients enrolled in cluster 2 had significantly more concerns about care personnel involved at 12 than at 3 months (p < 0.001).

### Effects of explanatory variables

We investigated the effects of three sociodemographic variables gender, age and education on each of the subscales of SUTAQ in patients in each of the Clusters. Results are presented in Table 3.

**Table 3:** Effects of explanatory variables on SUTAQ (significant coefficients).

Model	Unstandardized coefficients		T value	p-value
	B	Standard error		

CLUSTER 8 (PM/ICD)				

Determinants of Enhanced care subscale at 12 months				

Gender (Male)	9,67E + 01	3,77E + 01	2.56	**0.01**
**Determinants of Increased accessibility subscale at 12 months**				

Gender (Male)	0.12	0.05	2.63	**<0.01**
Low secondary education	–0.20	0.09	–2.24	**0.02**
High secondary education	–0.27	0.09	–2.92	**<0.01**
Higher education	–0.30	0.11	–2.73	**<0.01**
**Determinants of Care personnel concerns subscale at 12 months**				

Age	–0.002	0.001	–2.04	**0.04**
**Determinants of Satisfaction subscale at 12 months**				

Gender (Male)	0.09	0.03	2.71	**<0.01**
**CLUSTER 5 (COPD)**				

**Determinants of Care personal concerns subscale at 12 months**				

Higher education	0.87	0.38	2.25	**0.02**

The analysis of the effects of explanatory variables on SUTAQ subscales for cluster 2 (diabetes) and cluster 7 (CHF) did not show significant coefficients. In cluster 5 (COPD), the only significant coefficient shows that patients with higher education (p = 0.02) were more concerned about care personnel involved in the project. In cluster 8 (PM/ICD), there are significant coefficients referring to the male gender and its effects on the “Enhanced Care” subscale (p = 0.01), the “Increased accessibility” subscale (p = 0.009) and the “Satisfaction” subscale (p = 0.007). The results highlight that males seemed to be more satisfied than women, providing a higher positive evaluation of the introduction of telemedicine services. Moreover, in the same cluster, patients with lower secondary education (p = 0.02) perceived greater accessibility, whilst patients with higher secondary education (p = 0.003) and higher education (p = 0.006) perceived a lower level of accessibility. Finally, older patients were less concerned about care personnel (p = 0.04).

## Discussion

The evaluation of the patients’ perception showed a high level of acceptability of telemedicine services. This effect was extended over 12 months and in some cases the measure of acceptability increased between the 3 and 12 months assessments. Patients reported they were strongly satisfied with the management of the service and they thought it worked well. The service was acknowledged by patients as being an instrument capable of enhancing the care they were already receiving, as well as enabling people involved in their health management to better monitor their conditions. They reported that they were also encouraged to better monitor their own condition, having been given the opportunity to check their own parameters themselves. On this basis we hypothesize that patients felt that they were more involved and in control of their healthcare. With fewer concerns about their health and/or social care, these patients could then recommend the service to other people in a similar condition.

Patients perceived they were saving time because monitoring their health condition from home implied that the journey to health facilities was not always necessary. Furthermore, these patients felt that access to care was greater than usual, probably because they felt constantly reassured, being connected 24/7 to health and/or social care professionals through the Regional eHealth Centre. The exception in this case was for cluster 8 (PM/ICD) that only had relations with the usual reference clinician. Cluster 8 participants that received less than secondary school education (52%) present, on average, higher scores in the “Increased accessibility” subscale. It could be hypothesized that a level of education higher than “Elementary education” entails greater awareness about the functioning of the service and its limitations. The explanation of the service for cluster 8 occurred only once on delivery, while other clusters had the opportunity to receive continuous training and feedback from the operators of the Regional eHealth Centre. It is possible that patients who were more aware knew that clinicians were only periodically checking their data via the kit and they knew that the service was available only during working hours meaning that they perceived less benefits in terms of accessibility. On the other hand, less aware patients considered the kit as a 24/7 alarm and therefore they perceived a higher level of accessibility to health services.

Patients were not concerned about privacy issues, in particular they were not worried about sending clinical data remotely and did not report a negative impact on their emotional or physical life. The service was well perceived by patients, probably because telemonitoring had a positive influence on their perception of health care, with devices used at the patient’s home being compact and easy to use with no interference with their daily routine. Patients enrolled in cluster 7 (CHF) had a slightly more cautious approach to the service probably because they were enrolled after discharge from hospital following acute heart failure in the previous three months. After 12 months of intervention the results of the “Privacy and discomfort” subscale significantly improved in this cluster and aligned with other clusters.

Patients of all clusters showed a low level of concern about the health-care personnel involved in the telemedicine services. In particular those enrolled in cluster 8 presented with lower score on the “Care personnel concerns” subscale, which implies a strong positive view towards these aspect of telemonitoring. This is possibly due to the different form of service they received. These patients continued to have contacts only with the usual reference clinician during in-clinic follow up visits. Another aspect that should be considered is the fact that data transmission was partially automatized, removing concerns in patients about any issues arising between data measurement made by patients and data reception by the usual reference clinician. In cluster 8, older patients were less worried about care personnel. This may reflect the often reported tendency for older people to trust in clinicians with whom they are already familiar.

Patients enrolled in cluster 5 (COPD) showed more concerns than others about care personnel. These patients had problems carrying out measurements using pulse oximetry because the device sometimes did not work properly. In particular, patients with higher education were the most concerned about this issue, which probably reflects their greater awareness about chronic illness management and its implications.

Patients enrolled in cluster 2 (diabetes) had an enthusiastic approach to the service, but after 12 months of intervention they felt a little more concerned about the care personnel involved in the service. In particular some patients started to feel that the service partially interfered with the continuity of care they received. Initially their perception that the service could serve as a substitute differed from the other clusters, but after 12 months of intervention their results were similar. Like other clusters, after 12 months of intervention these patients felt less concerned about their health status. However, even if they felt safer, they did not value telemedicine as a total replacement for regular health or social care, as assessed by the “Kit as substitution” subscale and considered face-to-face consultation was still generally considered to be a more suitable solution.

## Conclusions

Telemedicine is seen as a potential integrated care solution to the problem of an ageing population affected by chronic disease and social care needs that will increase the demands of health care services. In this study, it has been shown that patients affected with different chronic illness accepted telemedicine services and reported a similar perception of it. No general difficulties were recorded and patients with the range of conditions studied gave a positive evaluation of the service, similar to other studies [27]. However, telemedicine services were not perceived as a total replacement for face-to-face consultations, but as a viable addition to usual care. Therefore, telemedicine should be considered an instrument capable of enhancing the self-management of chronic illness, guaranteeing satisfactory assistance and accessibility. There were no obstacles related to patient perception identified in this study that could preclude the implementation of the service, at least for the chronic conditions analysed in this study.

## Limitations

Results can be influenced by various factors that can increase risks of bias. First, although the SUTAQ is designed to be completed by an individual, it remains possible that some of the patients in the sample responded to the questionnaire by referring to relatives or other caregivers. Second, even if guideline translation rules have been followed and backward translation could have mitigated this factor, the translation of the SUTAQ questionnaire from English into Italian could have caused changes in the meaning of some items. Third no formal validation was conducted on the SUTAQ (eg. test-retest reliability) despite the rigorous methods adopted for the questionnaire translation and cultural adaptation and pretesting. Fourth, patients had difficulties in understanding the concepts of some items, in particular: “access to care” and “continuity of care”. Fifth, most patients were elderly and might have cognitive difficulties related to attention span and memory capacity and had difficulties in answering the questionnaire as reported by the operators of the Regional eHealth centre. Sixth, some patients had difficulties in answering using the Likert scale, probably because questionnaires were administered by telephone. Finally, the sample size was based on other outcomes, and the comparability between different clusters wasn’t planned, therefore the number of participants involved could not be adequate.

## Generalizability

In each cluster patients with different socio-demographic characteristics have been enrolled. Results for all clusters are similar suggesting a high level of generalizability.

## Registration number and name of trial registry

The following registration numbers identify the RENEWING HEALTH general trials:

Cluster 2 – Large-Scale Pilot in the Veneto Region: Life-long Monitoring of Diabetes Mellitus, ClinicalTrials.gov Identifier: NCT01569893; Cluster 5 – Large-Scale Pilot in the Veneto Region: Life-long Monitoring in COPD, ClinicalTrials.gov Identifier: NCT01513980; Cluster 7 – Large Scale Pilot in the Veneto Region: Remote Monitoring of Chronic Heart Failure, ClinicalTrials.gov Identifier: NCT01513993; Cluster 8 – observational study.
